# Pediatric abusive spine trauma: a review of whole-spine imaging essentials

**DOI:** 10.1007/s00247-026-06520-6

**Published:** 2026-01-31

**Authors:** Spencer Kriss, Alexandra Foust, Sumit Pruthi, Stephen Little, Asha Sarma

**Affiliations:** 1 Vanderbilt University School of Medicine, 1211 Medical Center Drive, Nashville, TN 37232 USA; 2 Monroe Carell Jr. Children’s Hospital, Nashville, TN USA; 3 Children’s Healthcare of Atlanta, Atlanta, GA USA

**Keywords:** Child abuse, Abusive head trauma, Abusive spinal trauma, Spinal subdural hematoma, Whole-spine MRI, Spinal ligamentous injury

## Abstract

Abusive spinal injuries, although often clinically silent, may require treatment and have medicolegal significance. Abused infants are prone to ligamentous injury of the craniocervical junction and upper cervical spine, which is often associated with brain hypoxic-ischemic injury. Although they are relatively common and highly specific for abuse, thoracolumbar spine injuries (e.g., thoracolumbar compression fractures and spinal extra-axial hemorrhages) are most often clinically occult. Additionally, they are often undetectable on skeletal survey and are frequently found in the absence of cervical spine injuries. Therefore, MRI screening of the whole spine is needed in selected cases of abusive trauma with potential for spinal injuries. This review discusses indications and summarizes recommendations for whole-spine imaging in cases of suspected child abuse. Imaging examples that emphasize the importance of whole-spine MRI in child abuse are provided. Vertebral fracture, spinal cord injury, spinal hemorrhage, and ligamentous and paraspinal soft tissue injury are discussed and illustrated.

**Graphical abstract:**

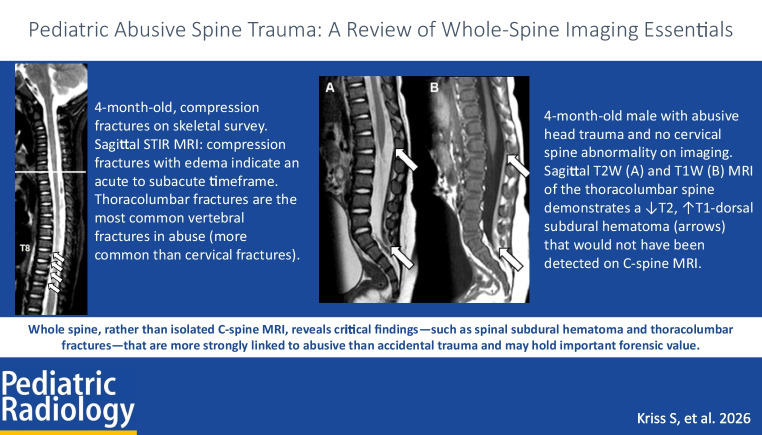

## Introduction

In children under 2 years of age, abusive head trauma (AHT) is responsible for 53% of serious or fatal traumatic brain injuries [1]. For affected patients, appropriate and timely imaging is crucial in characterizing all injuries for both clinical and forensic purposes, since no isolated injury or imaging finding is pathognomonic for child abuse. Rather, comprehensive multidisciplinary, multimodal clinical and imaging evaluation is utilized to determine the relative probabilities of abusive or accidental injury [2]. Much of the focus on neuroimaging in pediatric abusive trauma cases centers on AHT, including the cataloging and characterization of clinically significant injuries requiring immediate clinical intervention, such as subdural hematoma (SDH; the most frequently documented imaging finding), parenchymal injury, and skull fracture [3].

In the past, spinal MRI was not routinely performed, as the association of spinal injury with non-accidental trauma was underestimated, in both prevalence and importance [4, 5]. However, recent literature has demonstrated that spinal injury may occur in 30–40% of pediatric patients who have experienced abusive trauma [6]. Spinal injury is associated with poorer outcome measures in patients with AHT (longer intensive care stays, lower initial Glasgow coma scale score, and longer requirement for mechanical ventilation) [7–9]. Additionally, abusive spine injury that is missed upon initial evaluation may ultimately result in pain, deformity, mechanical instability, and clinical sequelae of spinal cord injury such as spasticity [7, 10]. Cataloging of spinal injuries as part of a comprehensive, whole body trauma evaluation may also be of forensic value, even when the spinal injuries are asymptomatic or do not require specific clinical intervention.

There is practice and protocol variation in the utilization of spine MRI in AHT [7]. Evidence supporting the inclusion of spinal MRI in child abuse imaging protocols has been mounting in recent years and is increasingly being addressed in literature and decision support guidelines.

This review discusses indications for whole-spine imaging in cases of suspected child abuse. Study selection and protocol recommendations are outlined. The benefits of whole-spine MRI in identifying overt and difficult-to-diagnose abuse-related spinal injuries are emphasized. Relevant literature is briefly reviewed. Lastly, MRI findings of abusive spinal trauma, including vertebral fractures, spinal cord injury, hemorrhage, ligamentous injury, and soft tissue injury, are illustrated to emphasize the importance of whole-spine imaging.

## The added value of whole-spine MRI in abusive trauma

Complete characterization of abuse-related injury is key to performing a comprehensive clinical and medicolegal evaluation. Physical exam findings in abusive spinal injury are often subtle or absent, particularly in preverbal children or those with altered consciousness due to head trauma [6]. Neurologic deficits are rare unless injury is severe, and physical signs like soft tissue swelling or deformity may go unnoticed. Thus, absence of physical exam findings does not rule out injury. Imaging findings are frequently crucial in assisting the medical and forensic teams to differentiate between abusive and accidental trauma [11–13]. If a pediatric patient who is suspected of being the victim of abuse presents with intracranial SDH, abnormal spine findings on skeletal survey, or localizing clinical exam findings of spinal injury, MRI of the spine has been recommended to search for spinal injuries [2, 3, 7].

Spinal injuries may include vertebral fractures, spinal ligamentous edema or disruption, extra-axial hemorrhage, and soft tissue edema [6]. Importantly, spinal soft tissue and ligamentous injuries and spinal SDH are encountered far more than spinal fractures in the setting of abuse [14]. Spinal MRI is more sensitive for detection of soft tissue, spinal cord, and ligamentous injuries than radiography and CT [7, 15]. Spinal MRI may also be helpful in establishing the mechanism of injury [9, 15].

Many available research studies of abusive spinal trauma are limited by lack of inclusion of thoracolumbar spine MRI; however, more recent publications have illustrated its increased yield (compared to cervical spine MRI) of significant findings such as spinal SDH and thoracolumbar fractures [7, 15–17]. For example, Karmazyn et al. found that 23% of whole-spine MRI examinations showed injuries (ligamentous, fracture, or subdural) localized to the thoracolumbar spine, and 51% of these major spinal findings identified were localized to the thoracolumbar spine [7]. Barriers to adopting routine whole-spine imaging may include additional scanner or procedural sedation time. Nevertheless, paralleling the growing body of evidence supporting this practice, recommendations for whole-spine MRI have increased and strengthened in guidelines over the last several years, as summarized in Table 1.

**Table 1:** Chronological summary of national and international guidelines addressing the role of spinal MRI in evaluation of pediatric patients with suspected abusive trauma

Source	Year of publication	Summary and key points
ACR Appropriateness Criteria	2017	• MRI of the cervical spine is “usually appropriate” in cases of suspected AHT and should be considered along with brain MRI [6] • Total spine MRI in suspected AHT “may be appropriate” on grounds that it may improve identification of thoracolumbar subdural hematoma
International Consensus Statement on AHT	2018	• Due to the extremely common nature of non-bony spinal abnormalities in AHT, whole-spine MRI “should be included in the diagnostic workup when there is evidence of intracranial injury” [1]
American Academy of Pediatrics (AAP)	2020	• MRI of the spine should be considered as part of the imaging assessment for AHT, emphasizing evidence on cervical spine injuries without discussing the use of whole-spine MRI [18]
Canty et al. on behalf of ENIGMA Child Abuse Working Group	2022	• Summarizes the available literature and encourages consistent recommendations for whole-spine imaging in children with AHT [3]
Consensus statement on the management of at-risk contact children	2023	• Imaging of the whole spine should be performed if there are positive findings on brain MRI [19]
European Society of Pediatric Radiology	2025	• Infants with suspected AHT and abnormal head CT or neurological findings should undergo brain and whole-spine MRI within 2 days to 5 days • The decision to image the spine in older children should depend upon the findings on neurological exam, skeletal survey, and head CT [2]
AAP	2025	• Cervical MRI should be performed when there is high clinical concern for spinal injury or identification of spinal injury on other imaging modalities • It is “prudent to strongly consider spinal MRI” in cases of AHT in which spinal injury would be “difficult to exclude clinically” or radiographically [20]. “MRI of the entire spine may also be valuable to evaluate for additional sites of spinal injury…or SDH” [20]
ACR Appropriateness Criteria	2025	• MRI complete spine without contrast “may be appropriate” in children <24 months or >24 months of age with physical exam or clinical findings suspicious for CNS injury or other injuries such as scalp bruises, hematoma, or skin injury to the head, neck, or spine. MRI only including the cervical spine is listed as “usually not appropriate” [21]

## MRI protocol for imaging of abusive spine trauma

At our institution, non-contrast whole-spine MRI is performed at the time of non-contrast brain MRI for patients with suspected AHT or other child abuse-related findings. MRI is ideally performed within 2 days to 5 days after clinical presentation for suspected child abuse; however, it may occasionally need to be delayed due to contraindications for MRI. Many children who have sustained abusive trauma with significant injuries require critical care, and many patients are imaged while sedated and on mechanical ventilation. Otherwise, general procedural sedation considerations apply, namely, that infants less than 2 months can usually undergo MRI without anesthesia (“feed and bundle” technique) and those over 2 months often require procedural sedation. Whole-spine rather than cervical spine MRI has been performed consistently in this context at our institution since 2022.

At minimum, the examination protocol should include axial and sagittal T1- and T2-weighted turbo spin echo (TSE) and sagittal short inversion recovery (STIR) sequences [7–9]. Sagittal T1-weighted FLAIR is preferred at 3-T field strength. Due to the significance of CCJ and upper cervical spine injuries, small field-of-view sagittal STIR images of the cervical spine and axial T2-weighted images of the CCJ may be performed [2, 9]. Sagittal T2 TSE may be preferred for spinal cord evaluation, with STIR preferred for assessment of the bone and ligaments. T1-weighted TSE imaging often suffers from flow artifacts that present a challenge when trying to identify extra-axial hemorrhage. Therefore, if axial T1-weighted imaging is performed, a gradient recalled echo sequence (e.g., VIBE or THRIVE) that is less susceptible to these artifacts may be preferred. At our institution, we also include sagittal T2*-weighted images to increase detection of blood products within the spinal canal. Table 2 reviews our institution’s spinal MRI protocol for abusive trauma evaluation. Including survey images, the average acquisition time for this protocol is approximately 50 min to 80 min, with a total session length of 90 min to 120 min for brain and whole-spine MRI.

**Table 2:** Sample spine MRI protocol for patients with suspected abusive injury

Sequence	FOV (cm)	Slice thickness (mm)	Slice spacing (mm)
Sag T2 TSE (best for cord evaluation)	Maximum 30	3	0.03
Sag STIR* (small FOV, C-spine)	Maximum 15	2	0.5
Sag T1 TSE	Maximum 30	3	0.03
Sag STIR*	Maximum 30	3	0.03
Sag T2*	Maximum 30	3	0.03
Ax T1 TSE (VIBE/THRIVE helpful to eliminate CSF flow artifact)	Maximum 16	3	1
Ax T2 TSE	Maximum 16	3	1

*STIR preferred over T2 FS TSE or mDixon for bone and ligament evaluation

## Review of spinal MRI findings in child abuse

### Extra-axial hemorrhage

Extra-axial (subdural and epidural) hemorrhage is a common injury in abusive spinal trauma [22]. Epidural hemorrhage is less common than SDH in abusive trauma and is more often spontaneous or associated with lumbar puncture [9]. Extra-axial spinal hemorrhages can vary in size. Intervention is rarely required, except in cases with cord compression and neurological decline. Importantly, this finding is rarely seen in accidental trauma (1–2% of cases) [17, 23].

Spinal SDH is considered one of the spinal MRI findings that are considered most specific for abusive trauma. In a 2019 series by Rabbitt et al. spinal SDH was associated with retinal hemorrhages, noncontact head injuries, and diagnosis of AHT, and was therefore felt to support a mechanism of severe acceleration and deceleration injury. Spinal SDH is often associated with AHT, seen in 67% of patients who underwent thoracolumbar imaging in one study [15].

Imaging of the whole spine is important to optimizing the sensitivity of MRI for intraspinal extra-axial hemorrhage. In their study of 148 suspected child abuse patients who underwent whole-spine MRI, Karmazyn et al. highlighted the importance of including the thoracolumbar spine on MRI by reporting that 23 of 34 SDH and two of three spinal epidural hemorrhages were isolated to the thoracolumbar spine [7]. In a study by Choudhary et al., 56% of spinal SDHs did not extend past the thoracolumbar junction, and including the thoracolumbar spine increased detection of SDH (with prevalence of 48% overall and 67% in the cohort with whole-spine imaging) [16].

Spinal SDH may result from redistribution of intracranial SDH or injury of spinal radicular veins [4]. Recent literature proposes a theory for the migration of intracranial hemorrhage into the spine due to injury of the myodural bridge complex. The myodural bridge complex is defined as a network of connective tissue connecting the suboccipital musculature to the cervical spinal dura mater. The physiologic purpose of this structure is to promote CSF flow and maintain a patent spinal canal during flexion and extension. In cases of AHT with extreme high-energy flexion and extension, rupture of the myodural bridge and traction on the intradural nerve roots and dentate ligaments may disrupt the dura-arachnoid interface and create a passage that allows subdural intracranial hemorrhage to migrate into the spinal subdural space [6, 24].

**Fig. 1:**
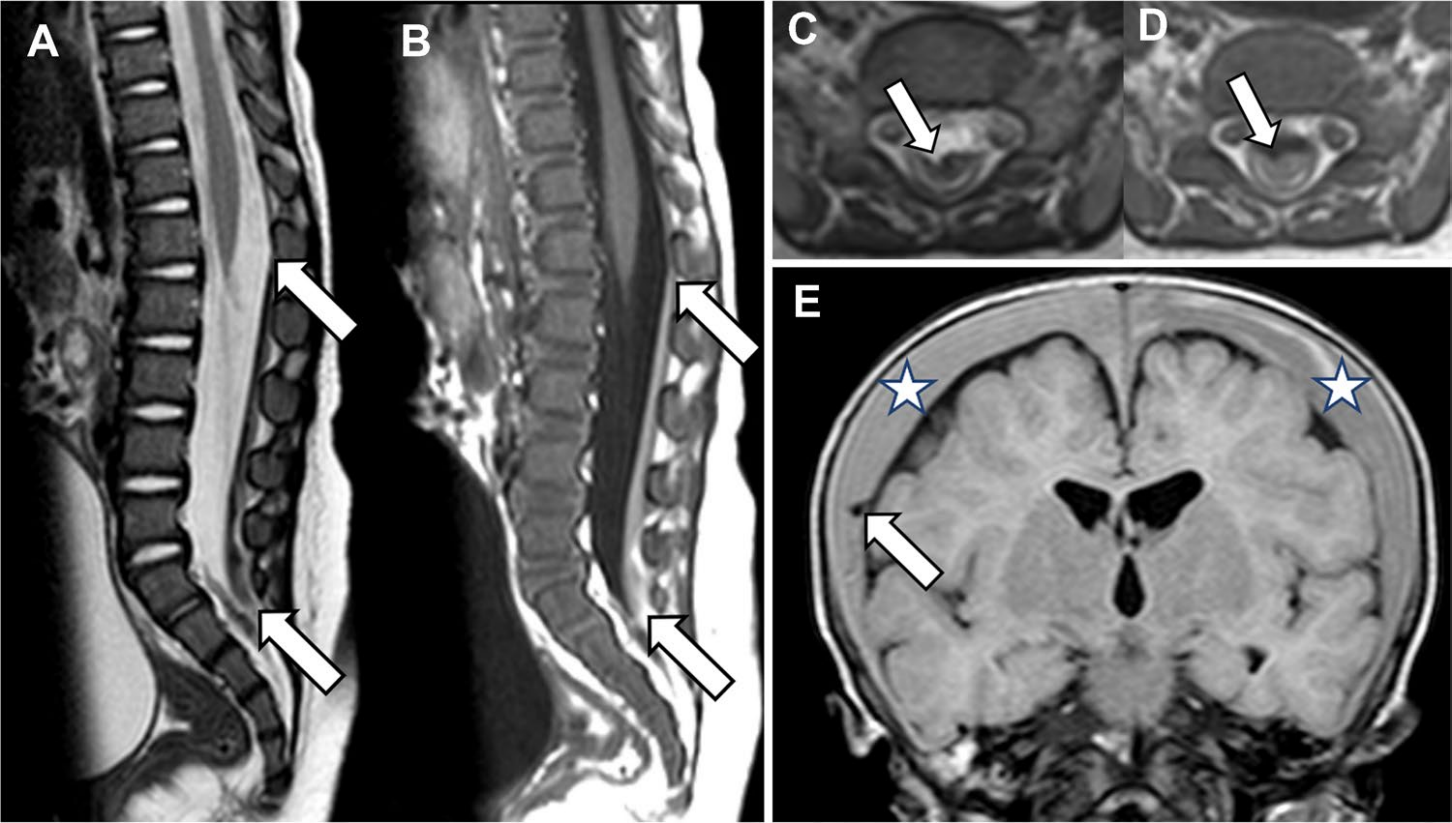
A 4-month-old male with abusive head trauma and thoracolumbar spinal subdural hemorrhage. Sagittal T2-weighted (**A**) and T1-weighted (**B**) MRI of the thoracolumbar spine demonstrates a T2-hypointense, T1-hyperintense dorsal SDH (*arrows*) extending from L1 to the distal thecal sac. Axial T2-weighted (**C**) and T1-weighted (**D**) MRI demonstrate a ventral indentation in the hematoma due to the presence of the midline dorsal septum, distinguishing it as subdural rather than epidural in location (the dorsal portion of an “inverted Mercedes Benz” sign). In this case, MRI of the cervical spine would have missed this critical finding. Coronal FLAIR brain MR image (**E**) shows bilateral convexity SDH (*asterisks*) and a torn or thrombosed bridging vein (*arrow*)

**Fig. 2:**
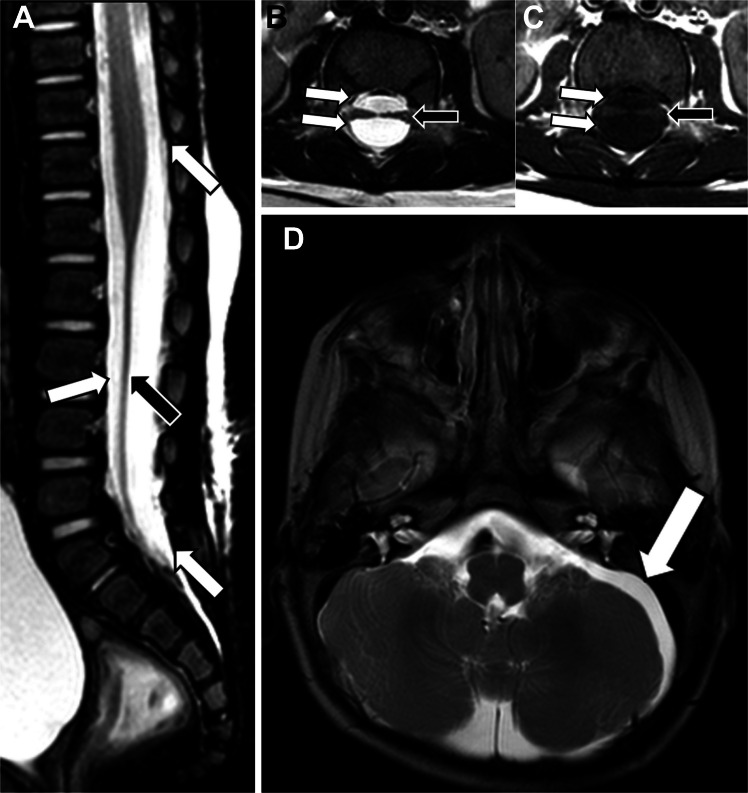
An 8-month-old male with suspected abusive head trauma and a thoracolumbar CSF-isointense extra-axial collection. Sagittal T2-weighted (**A**), axial T2-weighted (**B**), and T1-weighted (**C**) MR images of the thoracolumbar spine demonstrate a CSF-isointense dorsal and ventral fluid collection (*white arrows*) extending from T11 to the distal thecal sac, with compressed cauda equina nerve roots (*black arrows*). Axial T2-weighted image of the brain (**D**) demonstrates a CSF-isointense subdural collection in the left posterior fossa (*arrow*). No abnormality was seen in the cervical spine again highlighting the importance of whole-spine imaging (not shown)

The imaging appearance of spinal extra-axial hemorrhages can vary. Due to the unique spinal dural anatomical constraints of the lateral denticulate ligaments and the midline dorsal septum, spinal SDH often appears as two posterolateral collections and, sometimes, one anterior collection—this forms a so-called inverted Mercedes Benz sign [25]. In contrast, epidural hematomas are external to the dura mater and result in external compression of the thecal sac and effacement of the epidural fat [25]. Hemorrhage is often best identified on axial and sagittal T1-weighted and T2-weighted sequences as layering material that is variable in signal, but often T1-hyperintense and T2-hypointense relative to CSF (Fig. 1). It is important to remember that spinal subdural collections can resemble CSF (Fig. 2) [4]. Although the reason for this is incompletely understood, arachnoid tearing (as is thought to be a factor in CSF-isointense intracranial hygromas in children with AHT) or redistribution of CSF-isointense fluid from intracranial subdural collections may contribute to this phenomenon [15, 24, 26].

**Fig. 3:**
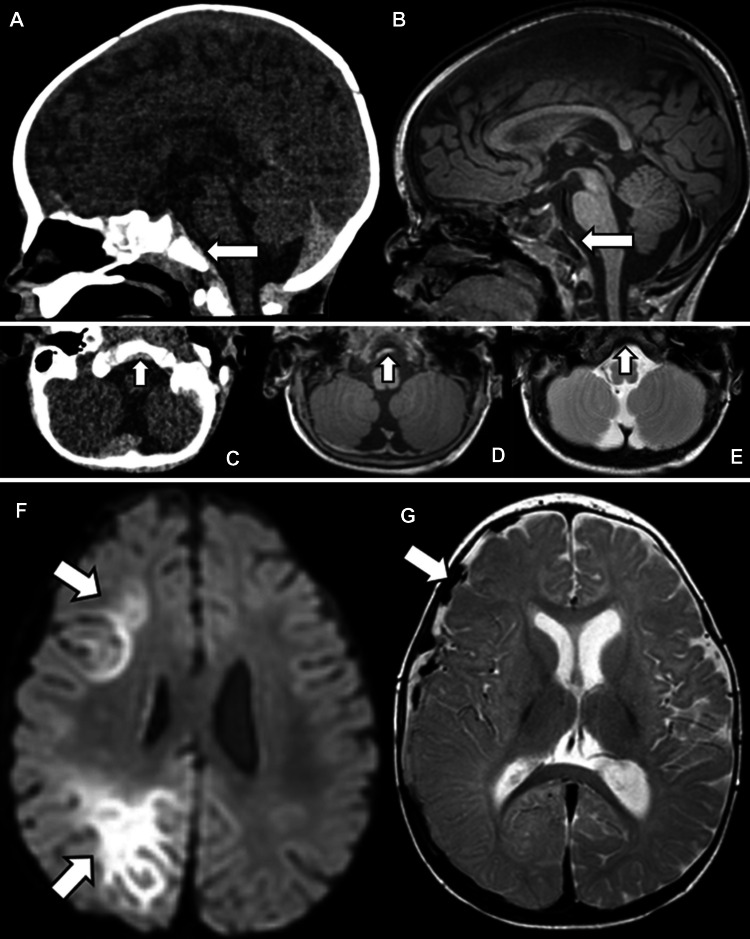
A 6-month-old male with abusive head trauma and retroclival hematoma on CT and MRI performed 3 days later. Sagittal CT (**A**) and T1-weighted MRI (**B**) demonstrate posterior fossa SDH, including hyperattenuating, T1-hyperintense retroclival hematoma (*arrows*). Axial CT (**C**) and T1-weighted (**D**) and T2-weighted (**E**) MR images of the posterior fossa show the hyperattenuating, T1-hyperintense, T2-hypointense hemorrhage (*arrows*). Axial diffusion-weighted (**F**) and T2-weighted (**G**) MRI of the brain show hypoxic injury (*arrows* in **F**) and mixed signal intensity SDH with injured bridging veins (*arrow* in **G**). CT and MRI are often complementary in the detection of small retroclival hemorrhages, and evaluation of both modalities can increase diagnostic confidence

Retroclival hematomas are commonly associated with hypoxic brain injury and abusive injury to the CCJ (e.g., tectorial membrane injury) and can be epidural or subdural in location [27]. These collections can be subtle, and it is important for the radiologist to scrutinize the retroclival region to improve detection. An important pitfall to recognize is that hyperattenuating unossified odontoid cartilage may be seen in a similar location and mimic hemorrhage on CT. It is important to use narrow “subdural windows” on axial and sagittal images to differentiate hyperattenuating retroclival hemorrhage from the adjacent hyperattenuating clival cortical bone. Additionally, retroclival hematoma may be difficult to detect in the setting of herniation with effacement of the prepontine cistern. Finally, differentiating retroclival hematoma from the cortex of the adjacent clivus, which itself can be T2-hypointense with blooming artifact, can be difficult on MRI. Although MRI may be more sensitive than CT overall for retroclival hemorrhage, the two modalities can be complementary (Fig. 3) [27].

### Spinal ligamentous and paraspinal soft tissue injuries

In the cervical spine, ligamentous injuries are the most common finding of abusive trauma and are best visualized with MRI. PD-weighted imaging in coronal and sagittal planes or isotropic PD-imaging may be helpful for visualization [12, 28–30]. Cervical spinal ligamentous injury is more commonly associated with abusive than accidental trauma [16]. Spinal ligamentous injury is a poor prognostic factor in children with AHT in combination with intracranial hemorrhage, diffuse axonal injury, HII, and arterial stroke [8]. One potential explanation for HII is that CCJ mechanical instability due to ligamentous injury can lead to spinal cord and brainstem compression, resulting in apnea and irregular breathing that decrease brain oxygen supply [16, 31, 32]. Cervical nerve root injury causing diaphragmatic paralysis may also contribute to anoxic injury [33].

**Fig. 4:**
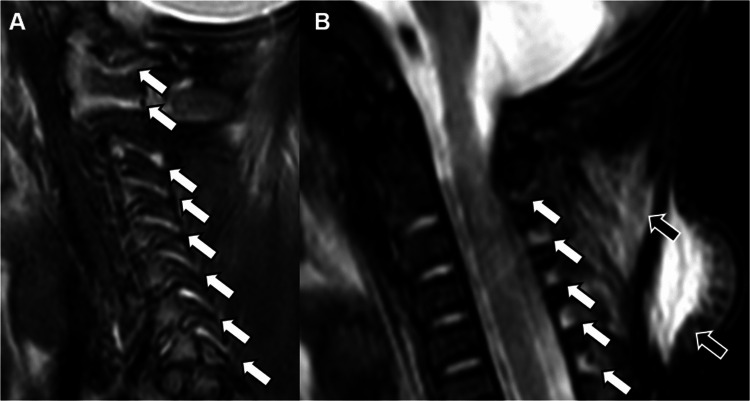
A 3-month-old male with CCJ and upper cervical spine ligamentous and paraspinal edema. Sagittal off-midline T2 mDixon water-only image (**A**) demonstrates multilevel facet capsular edema extending from the atlantooccipital joint to T1-T2 (*arrows*). Midline image from the same sequence (**B**) demonstrates multilevel interspinous ligament (*white arrows*), nuchal ligament, and subcutaneous edema (*black arrows*). Posterior paraspinal soft tissue edema is relatively common in imaging from abusive trauma and may be partially attributable to prolonged immobilization or post-resuscitation fluid shifts in some cases

The posterior ligament complex (PLC) contains the supraspinous and interspinous ligaments, articular facet joint capsule, and the ligamentum flavum [31]. At the level of the CCJ, the PLC is comprised of the nuchal ligament, atlantoaxial and atlantooccipital membranes which are thin structures posterior to the dura, interspinous ligaments, and capsular ligaments of the occiput-C1 and C1-2 articulations [31]. The anterior ligament complex is comprised of the apical and alar ligaments of the dens, the anterior and posterior longitudinal ligaments, the cruciform ligament, and the tectorial membrane [31]. In infants, posterior ligamentous injury is common in AHT, as the posterior ligaments are inherently less stable than the anterior ligaments. There is an increased risk for CCJ instability in patients with trisomy 21, juvenile idiopathic arthritis, upper respiratory infection, or skeletal dysplasia [34]. Posterior ligamentous injuries are often characterized by abnormal fluid signal or frank ligamentous disruption, with interspinous distance widening and adjacent paraspinal edema on MRI (Fig. 4) [6]. It is important to distinguish this edema from normal T2 hyperintense vessels and other structures. Hyperintense signal of the posterior atlantoaxial and atlantooccipital membranes on fluid-sensitive sequences suggests acute ligamentous injury [9, 31]. Small field-of-view imaging of the CCJ and upper cervical spine can improve the characterization of these injuries.

Other findings associated with spinal ligamentous injury include increased joint fluid when there is damage to the capsular facet (Fig. 4). Mild distraction injury causing effusion, with or without joint widening, is commonly seen in AHT [31, 35]. Although unstable injury that necessitates surgical intervention is rare, severe injury to the posterior spinal ligaments may cause significant instability that necessitates surgical management [36].

Posterior paraspinal muscular and nuchal ligament edema are very common, affecting more than one-third of patients with AHT [9, 35]. MRI has been shown to have a high false positive rate due to posterior paraspinal edema related to post-resuscitation fluid shifts [9, 36]. In our own institutional experience, both visible capsular fluid (without distraction) and posterior paraspinal edema have been found very commonly in infants undergoing spinal MRI for suspected abusive injury, regardless of the ultimate clinical determination of abusive versus accidental injury. The trauma population comprises a large majority of infants undergoing cervical spine MRI at our institution, so it is difficult to compare the relative incidence to that in patients without a trauma history. We hypothesize that, in some cases, posterior paraspinal edema and visible capsular fluid may be at least partially attributable to factors such as prolonged cervical immobilization (which has been found to lead to soft tissue injuries such as decubitus ulcers) and fluid shifts [36]. However, formal studies comparing the incidence of these findings in infants undergoing cervical spine MRI for abusive trauma versus non-traumatic indications, which could clarify the true attribution of these findings, have not been performed to date. Therefore, based on available evidence, visible capsular fluid should be presumed to be due to traumatic injury in the setting of suspected abusive trauma at this time.

### Vertebral fractures

Vertebral fractures in infants or non-ambulatory children should raise strong concern for abuse, especially when the reported mechanism is inconsistent or there are additional injuries (e.g., rib fractures, intracranial hemorrhage, retinal hemorrhages) [10]. In cases where spinal fracture has been identified, there is significant correlation with associated intracranial injury as well as increased incidence of additional acute and healing skeletal fractures (as identified on skeletal survey), particularly in fatal cases [37, 38]. Although they are less common in abusive trauma than thoracolumbar fractures, cervical spine fractures are more likely to be associated with neurological findings [6, 10].

Infants are at higher risk for injury to the CCJ and upper cervical spine due to a relatively high head size to body ratio; the large head of an infant can comprise almost a third of its total body weight [31]. Additional risks for injury to the CCJ and upper cervical spine include weak neck muscular support, higher ligamentous and capsular and synovial laxity, and other bony and ligamentous anatomic features [10, 39]. These physical characteristics lead to hypermobility of the cervical spine with the high mechanical energy imparted in cases of AHT. As a result, cervical spinal fractures are common in cases of abusive trauma in infants with impulse loading due to shaking [6]. Imagers should be careful to utilize pediatric reference values for assessment of the cervical spine and CCJ and be aware of normal variants such as cervical spine “pseudosubluxation,” which can be confirmed by the absence of bony or soft tissue edema on MRI in equivocal cases [34, 40]. CCJ injuries may include fractures of the occipital bone and/or the C1 and C2 vertebrae, and atlanto-occipital or atlanto-axial subluxation or distraction [10].

**Fig. 5:**
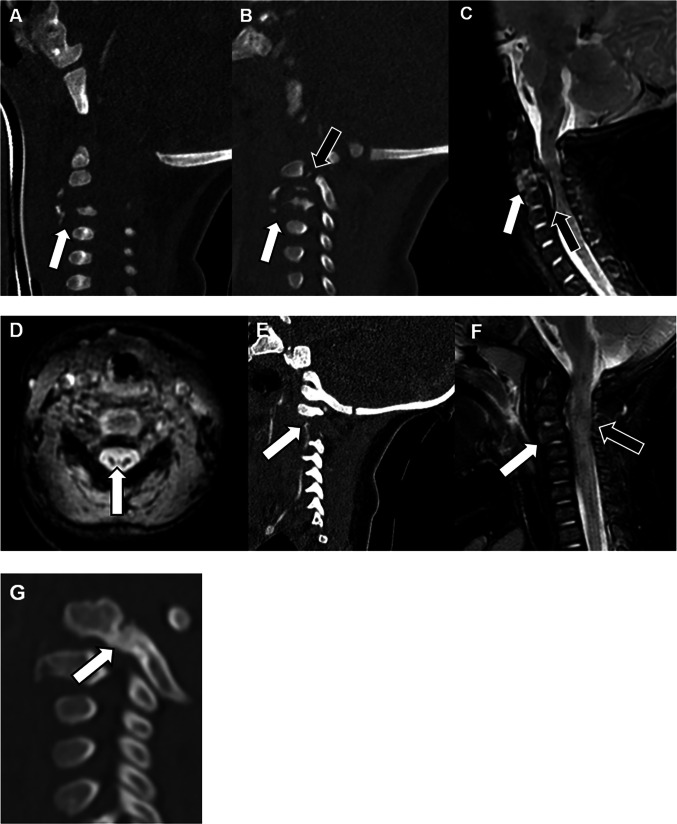
A 2-month-old female with severe abusive head and spine trauma, with follow-up cervical spine imaging at 4 months and 7 months of age. Sagittal CT images in midline (**A**) and off-midline (**B**) demonstrate extensively comminuted C3 burst (*white arrows*) and displaced C2 pedicle (*black arrow*) fractures, with anterolisthesis of C2 on C3. The C2 pedicle fractures were bilateral (contralateral fracture not pictured). Sagittal STIR MRI (**C**) shows edema associated with the burst fracture (*white arrow*) and uplifting of the posterior longitudinal ligament (*black arrow*) leading to spinal cord compression. Axial T2* MRI (**D**) demonstrates blooming artifact in the spinal cord due to intramedullary hemorrhage. Sagittal CT angiography maximum intensity projection image of the neck (**E**) shows left vertebral artery dissection (*arrow*). Right vertebral artery dissection was also present (not shown). Sagittal T2-weighted mDixon water-only image (**F**) obtained at 4 months of age, following non-operative treatment with immobilization, demonstrates resolution of acute findings including C3 vertebral body edema (*whitearrow*) and posterior longitudinal ligament injury, as well as mildly increased caliber and T2-hyperintense signal of the spinal cord (*blackarrow*). Sagittal CT image (**G**) at 7 months of age demonstrates partial healing of the C2 pedicle fracture (*arrow*)

If a vertebral fracture is identified on imaging, particularly a cervical fracture, there should be heightened concern for spinal cord and vascular injury. Burst fractures should prompt thorough neurological evaluation for cord injury from displaced fracture fragments (Fig. 5) [41]. Injuries associated with burst fractures can include damage to the posterior longitudinal ligament, epidural hematoma causing cord compression, and intramedullary hemorrhage.

**Fig. 6:**
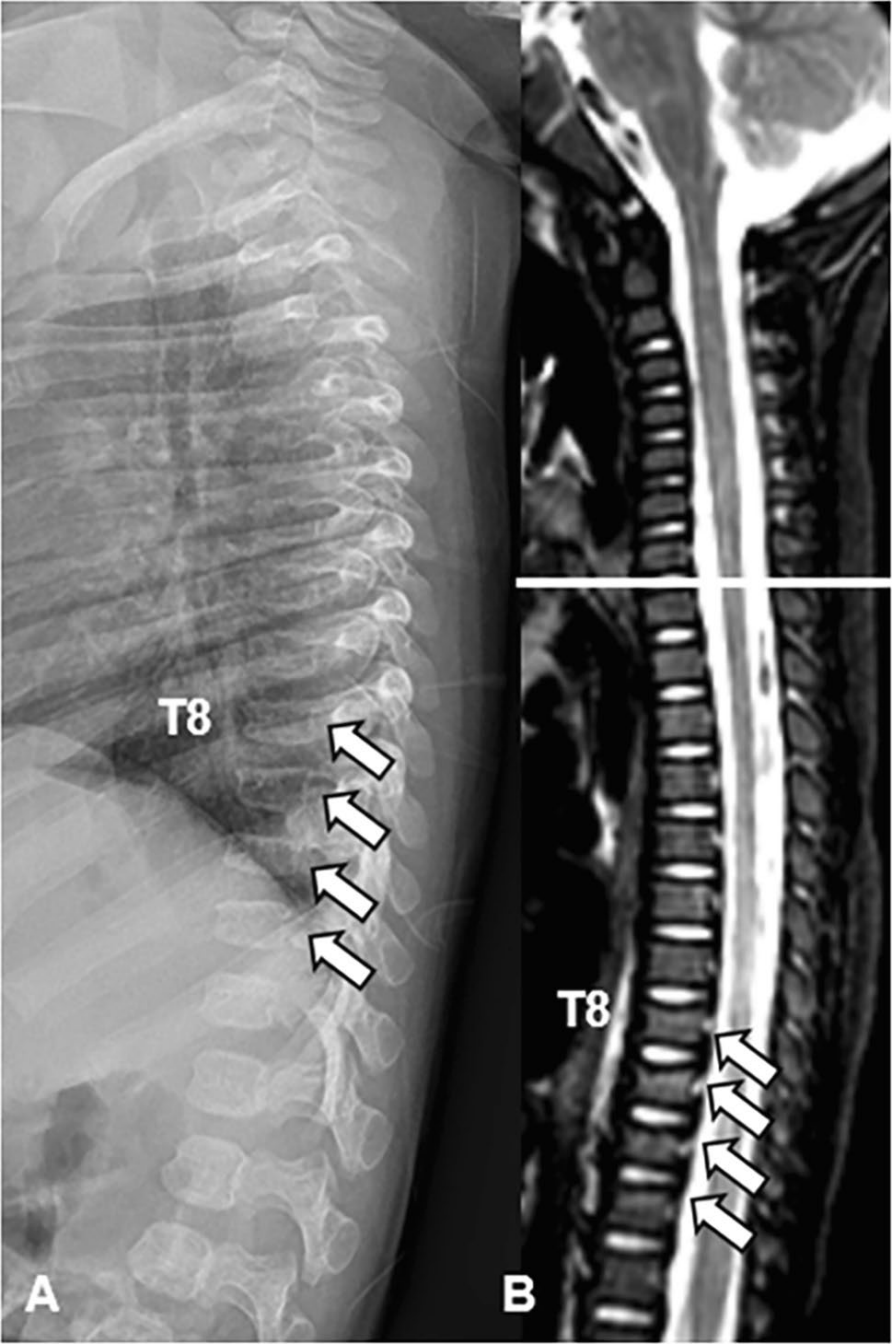
A 4-month-old male with abusive thoracolumbar compression fractures. Lateral image from a radiographic skeletal survey (**A**) demonstrates vertebral body height loss due to compression fractures of T8-T11 (*arrows*). Sagittal STIR MR image (**B**) demonstrates edema in the affected vertebral bodies (*arrows*), indicating an acute to subacute timeframe. Although there were multiple healing rib fractures (not shown), no head injuries or cervical spine injuries were present in this case (note normal cervical spine appearance above *white line* in **B**), highlighting the importance of whole-spine MRI

Potential mechanisms of compression fractures (again, thoracolumbar compression fractures are the most common spinal fracture seen in abusive trauma) include axial-loading, forceful flexion-extension, or shaking, especially when a child is held by the torso or lower limbs [37]. Karmazyn et al. found that 9 of 11 vertebral fractures in their series were isolated to the thoracolumbar spine, including five that were not identified on skeletal survey [7]. Therefore, ensuring that imaging includes the whole cervical, thoracic, and lumbar spine is crucial to identifying fractures that would have been missed if only cervical MRI had been performed (Fig. 6).

**Fig. 7:**
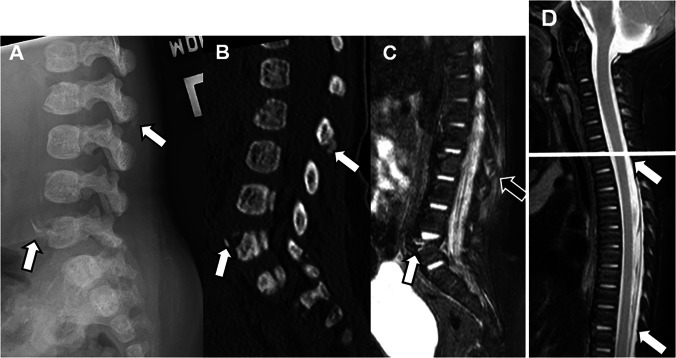
A 4-month-old female with abusive head trauma, retinal hemorrhages, and thoracolumbar spine injuries. Lateral lumbar spine radiograph from skeletal survey (**A**) and sagittal CT (**B**) performed for better characterization demonstrate displaced fractures of the L2 spinous process and L5 superior endplate (*arrows*). Sagittal STIR (**C**) MR image demonstrates edema associated with the L5 fracture (*whitearrow*) and paraspinal and multilevel interspinous ligament edema (*blackarrow*). Sagittal STIR of the upper spine (**D**) demonstrates a thin CSF-isointense epidural collection extending inferiorly from the T1-T2 disc level (*whitearrows*). Note that MRI of the cervical spine only (above *whiteline* in **D**) would not have included any of the spinal injuries illustrated here

Thoracolumbar compression fractures are usually clinically silent and more commonly appear at older ages [20]. In a systematic review, Kemp et al. noted that thoracolumbar injury presented at a median age of 13.5 months, compared to 5 months for cervical injury [42]. While thoracolumbar compression fractures may be identified upon skeletal survey, specifically lateral radiographs, MRI is recommended for identification of additional fractures and comprehensive evaluation of fracture characteristics (e.g., edema) and associated soft tissue injuries (Fig. 7).

MRI is sensitive for marrow edema, adjacent soft tissue swelling, and associated spinal ligamentous injury [7, 43]. Unlike compression fractures, vertebral contusions are only distinguishable through bone marrow edema on MR. Fluid-sensitive sequences are particularly helpful for detecting subtle end plate edema in cases with minimal vertebral body height loss. MRI detection of marrow edema is also useful for distinguishing normal variants from thoracolumbar compression fractures that can be equivocal on initial skeletal survey.

### Spinal cord and nerve root injuries

The reported prevalence of spinal cord injury (SCI) in abusive trauma depends heavily on whether imaging (especially MRI) was done, whether imaging was of the cervical or whole spine, the timing of imaging with respect to injury, and how spinal injury is defined in the study (i.e., variable inclusion of ligamentous injury, hemorrhage, vertebral fracture, and spinal cord injury). However, SCI defined as parenchymal damage or a clinically referable neurological deficit appears to be rare—often under 10% in the studies that report it [44]. Mechanisms of SCI in AHT include high-energy repetitive flexion/extension, traction injury, and cord ischemia [6].

Utilization of MRI has significantly increased the identification of occult SCI in AHT [12]. Historically, when radiographs were the primary imaging modality for suspected child abuse, SCI without radiographic abnormality (SCIWORA) was a well-documented phenomenon [45]. The previously described biomechanical characteristics of the infant CCJ predispose to transient malalignment and SCI without a visible fracture or persistent malalignment on radiography. MRI findings of abusive SCI may include compression, laceration, contusion, intramedullary hemorrhage, and transection (Fig. 5) [9, 10, 46]. There may be associated nerve root avulsions. Although MRI is the most sensitive imaging modality for intramedullary hemorrhage, it may be challenging to identify due to CSF pulsation and motion artifacts [28, 47]. Imaging performed in a delayed fashion with respect to the traumatic incident may show signs of a previously missed SCI, such as myelomalacia or syrinx [10].

SCI is often, but not always, associated with spinal extra-axial hemorrhage [48]. Identification of SCI plays a significant role in supporting the diagnosis of AHT, including correlation in follow-up neuropathology in postmortem cases; upon pathologic examination, injury of the spinal nerve roots was seen almost three times as often in AHT when compared to accidental head trauma [49]. It has been proposed that the mechanism in children with SCI without typical intracranial findings of AHT may be different than that in children with AHT. Although the mechanism remains unknown, grasping and shaking of the patient’s head have been proposed [48].

## Conclusion

Accurate cataloging of spinal injuries in child abuse is critical despite the fact that many of these injuries do not require clinical management changes—these injuries are often noteworthy for their medicolegal significance. Spinal bone and soft tissue injuries are often asymptomatic and clinically and radiographically occult, highlighting the importance of spinal MRI in child abuse evaluation. Whole-spine MRI increases the detection of key findings such as spinal SDH and thoracolumbar vertebral fractures, which carry significant relevance due to their relatively high specificity for abusive rather than accidental mechanisms. Recently, recommendations in clinical guidelines have increasingly reflected a growing body of evidence for whole-spine rather than cervical spine MRI in cases of suspected child abuse.

## Data Availability

No datasets were generated or analysed during the current study.
